# Increasing the proportion of plasma MUFA, as a result of dietary intervention, is associated with a modest improvement in insulin sensitivity

**DOI:** 10.1017/jns.2019.29

**Published:** 2019-11-29

**Authors:** I. Johns, G. Frost, A. Dornhorst

**Affiliations:** Nutrition and Dietetic Research Group, Imperial College London, London, UK

**Keywords:** Fatty acids, MUFA, Insulin sensitivity, Insulin resistance, Type 2 diabetes mellitus, %NEFA_total_, percentage of total NEFA, FA, fatty acid, GI, glycaemic index, HGI, high glycaemic index, HM, high MUFA, HS, high-SFA, LGI, low glycaemic index, PL, phospholipid, RISCK, Reading, Imperial, Surrey, Cambridge and Kings, Si, insulin sensitivity index, T2DM, type 2 diabetes mellitus

## Abstract

The effect of modifying dietary fatty acid (FA) composition on insulin sensitivity remains unclear. We aimed to investigate whether changes in plasma phospholipid (PL) FA composition, as a result of dietary intervention, correspond with changes in insulin sensitivity. The RISCK study was a 6-month randomised controlled dietary intervention study, which assessed the effect of modifying dietary fat and the glycaemic index (GI) of carbohydrates on insulin sensitivity. Total NEFA levels, fasting plasma PL FA profiles and an insulin sensitivity index (Si), derived from intravenous glucose tolerance minimal-model analysis, were available from 533 participants, all at elevated risk of type 2 diabetes. Bivariate correlations between changes in saturated PL FA (SFA), MUFA (as a percentage of total plasma NEFA) and changes in Si were assessed according to treatment group. Age, sex, ethnicity, percentage change in body mass and change in dietary GI were controlled for. Increasing total NEFA concentration was associated with worsening Si (*r −*0·152; *P* = 0·001). In the high-MUFA/low-GI diet group, change in PL-MUFA was positively and independently associated with change in Si (*r* 0·297; *P* = 0·002). Among MUFA, change in oleic acid (18 : 1) was most strongly correlated with change in Si (*r* 0·266*; P* = 0·005), as was change in minor FA 24 : 1 (*r* 0·244; *P* = 0·011) and 17 : 1 (*r* 0·196; *P* = 0·042). In the high-SFA/high-GI group, change in SFA concentration was not significantly associated with change in Si. In conclusion, increases in the proportion of plasma PL-MUFA following a high-MUFA dietary intervention were associated with improvements in insulin sensitivity.

Type 2 diabetes mellitus (T2DM) is a significant cause of morbidity and mortality and the prevalence of the disease is anticipated to increase dramatically as a result of the obesity epidemic^(1)^. There is substantial evidence that energy-dense, high-fat diets are diabetogenic and dietary modification targeted at reducing total energy and fat intake is an effective strategy in the prevention of T2DM, with superior outcomes to pharmacological interventions^(2,3)^. However, there is uncertainty regarding the effect of modifying dietary fat composition on insulin sensitivity.

Results from randomised trials have suggested that substituting saturated fat for unsaturated fat improves insulin sensitivity^(4,5)^. However, another randomised study failed to demonstrate any benefit of reducing dietary saturated fat in a weight-stable obese European population with the metabolic syndrome^(6)^. Thus, the optimum dietary approach with regard to fat and T2DM prevention remains unclear. There is also evidence that individual NEFA influence insulin sensitivity both positively and negatively, and to varying degrees^(7)^. However, it is currently unclear to what extent dietary fatty acid (FA) composition may affect one's propensity to developing impaired glucose tolerance through changes in insulin sensitivity.

To provide evidence-based nutritional guidelines on dietary fat and T2DM prevention, controlled intervention studies are required. RISCK (Reading, Imperial, Surrey, Cambridge and Kings) was a large intervention study, which manipulated dietary fat and carbohydrate composition in free-living individuals^(8,9)^. Results from controlled feeding studies have established that plasma NEFA composition reflects dietary fat intake^(10)^. It follows that any beneficial or harmful effects of individual plasma FA or FA groups on insulin sensitivity should be modifiable by diet. Initial results from the RISCK study indicated that isoenergetic replacement of dietary saturated fat with monounsaturated fat had no effect on insulin sensitivity^(11)^. However, it has not been yet considered whether changes in the proportion of individual plasma SFA and MUFA were associated with changes in insulin sensitivity. We aimed to investigate whether changes in the plasma phospholipid (PL) FA composition, as a result of the dietary interventions implemented in the RISCK study, correspond with changes in insulin sensitivity.

## Method

RISCK was a large randomised controlled dietary intervention study conducted across five leading UK nutritional research centres: Reading, Imperial, Surrey, Cambridge and Kings. The study was performed in free-living participants at elevated risk of metabolic disease, and assessed the effect of modifying type of dietary fat and the glycaemic index (GI) of carbohydrate on metabolic risk factors. The study design has been described in full elsewhere^(8,11)^.

### Subjects

Ethical approval for the RISCK study was obtained from the National Research Ethics Service, and written informed consent was given by participants. Men and women aged 30–70 years who met selection criteria (Supplementary Table S1) were recruited from the general population. Participants were screened to identify individuals at increased metabolic risk but below a level warranting clinical intervention.

### Study design

A total of 720 participants were enrolled and randomised to five dietary intervention groups by a computerised system designed to balance age, sex, waist measurement and HDL-cholesterol between groups (Supplementary Table S2). In all, 548 participants completed the study (23·9 % dropout rate). The study provided isoenergetic dietary substitutions for fat- and carbohydrate-rich foods while allowing subjects to eat *ad libitum*. Key exchangeable sources of dietary fat (cooking oils, spreads and margarine) and carbohydrates (e.g. pasta, rice, bread, cereal) were substituted for study foods with measured FA profiles and GI. In order that diets were isoenergetic, carbohydrate levels were modified to ensure that the changes to fat intake were balanced by carbohydrate intake.

All subjects received the high-SFA and high-GI (HS/HGI) reference diet for a 4-week run-in period, which represented the average UK diet (about 18 % saturated fat; GI of 63). After a run-in period, baseline measurements were performed and one of the five trial diets prescribed for 24 weeks. Nutrition diaries were completed at screening, end of run-in, 12 weeks and 24 weeks, from which dietary fat composition was calculated. The food exchange model successfully achieved the targets for dietary fat and carbohydrate in each of the five diets^(9)^.

At the beginning and end of the dietary intervention, baseline characteristics were assessed. Fasting blood samples were collected for plasma PL FA profiles and a short intravenous glucose tolerance test performed from which an insulin sensitivity index (Si) was derived via minimal modelling. Total plasma NEFA concentrations were assessed with an enzymic colorimetry assay and PL FA concentrations were quantified by a GC method as described in full elsewhere^(9)^. Individual FA and FA subclasses, where reported, refer to plasma PL FA. A complete list of PL FA analysed is available in Supplementary Table S3.

### Data analysis

Statistical analysis was performed with IBM SPSS statistics software. Correlations are presented as Pearson's *r* values, with corresponding *P* values. Si results were assigned standardised values. Results with *z*-score > ±3·3 were defined as outliers^(12)^ and removed from analysis.

Bivariate correlations of baseline (post-run-in period) total plasma NEFA concentration, total PL SFA, unsaturated fatty acids, MUFA, PUFA, *n*-3 PUFA and *n*-6 PUFA and Si were assessed for the whole cohort.

Previous work by Moore *et al*.^(9)^ has demonstrated that there were significant changes in the plasma PL FA profiles between groups following the 6-month dietary intervention. RISCK study subjects who consumed high-MUFA (HM) diets had significantly elevated plasma PL MUFA levels (% total FA) when compared with the other intervention groups combined (HS/HGI, low-fat/HGI, low-fat/LGI), following the dietary intervention^(9)^. In accordance with this previous analysis, we report the association between change in the percentage of the total FA pool comprising of plasma PL MUFA, and change in Si, for all subjects receiving HM diets and for the two individual HM subgroups (high monounsaturated fat, high GI (HM/HGI) and high monounsaturated fat, low GI (HM/LGI)). The association between change in the proportion of individual MUFA and change in Si was also assessed within the HM subgroups. We also assessed whether the influence of changes in individual MUFA concentrations was additive. Partial correlations were performed to adjust for change in GI between run-in and post-intervention to assess the unique effect of the change in MUFA concentration on Si. Additionally, linear regression was performed to adjust for age, sex, ethnicity and for percentage change in body mass across the study period. β Coefficients, where reported, are standardised. When changes in individual fat subtypes have been assessed against change in Si, changes in other subgroups were controlled for except for where the variables were excessively collinear. Excessive collinearity was defined as a variance inflation factor of >10^(13)^. Binary logistic regression analysis was also performed, with change in Si categorised as positive and negative, with the same variables entered as per linear regression analysis. Additionally, change in reported MUFA intake per kJ consumed was correlated with change in plasma PL levels from post-run-in to the end of the dietary intervention.

## Results

Of the 549 participants who completed the RISCK study, intravenous glucose tolerance test results and FA profiles were available for 533. Subjects' baseline characteristics are shown in Table 1.

**Table 1.** Baseline characteristics of study participants (Mean values and standard deviations; numbers of participants and percentages; medians and ranges)

	Male (*n* 227)				Female (*n* 306)			
Characteristic	Mean	sd	*n*	%	Mean	sd	*n*	%
Age (years)								
Median	54				52			
Range	40				40			
Ethnicity								
White			187	87·2			236	77·1
South/South East Asian			21	9·3			31	10·1
Black			12	5·3			28	9·2
Far East			1	0·4			2	0·7
Other			4	1·8			7	2·3
BMI (kg/m^2^)	28·5	3·8			28·8	5·3		
Waist (cm)	102·2	10·4			94·2	12·1		
Average insulin (pmol/l)	68·4	32·0			63·1	29·5		
Average glucose (mmol/l)	5·7	0·5			5·5	0·6		
Insulin sensitivity (×10^−4^ ml/μU per min)	2·9	1·9			3·3	2·1		
AIRg (ml/μU per min)	489·4	364·5			466·5	339·5		
Systolic blood pressure (mmHg)	134·3	15·1			125·5	15·3		
Diastolic blood pressure (mmHg)	82·5	9·0			77·4	9·0		
Total cholesterol (mmol/l)	5·5	0 9			5·6	1·0		
HDL-cholesterol (mmol/l)	1·3	0·3			1·5	0·4		
TAG (mmol/l)	1·7	0·8			1·4	0·6		
Smokers			17	7·5			16	5·2
Anti-hypertensive medication			44	19·4			51	16·7
HRT			–	–			34	11·1
Oral contraceptive			–	–			10	3·3
Thyroxine			2	0·9			22	7·2

AIRg, acute insulin response to glucose; HRT, hormone replacement therapy.

Total plasma NEFA concentration at baseline was inversely correlated with Si (*r* −0·108; *P* = 0·013). Total SFA was also inversely associated with Si (*r* −0·106; *P* = 0·014) as were total unsaturated NEFA (*r* −0·088; *P* = 0·042), total PUFA (*r* −0·098; *P* = 0·041) and *n*-6 PUFA (*r* −0·093; *P* = 0·032). There was no association between total MUFA or *n*-3 PUFA and Si.

Change in total plasma NEFA concentration across the study period within the whole cohort was inversely associated with Si (*r −*0·152; *P* = 0·001). In the high-saturated fat group, changes in SFA (percentage of total NEFA; %NEFA_total_) were not associated with a change in Si. Intervention group was not a significant interacting factor when included in stepwise regression analysis. Plasma PL FA levels according to intervention group (percentage of total FA) at baseline (post-run-in) and post-intervention are shown in Table 2.

**Table 2.** Plasma phospholipid fatty acid levels according to intervention group (percentage of total fatty acids) at baseline (post-run-in) and post-intervention (Mean values and standard deviations)

	Total cohort (*n* 533)		HM (*n* 221)		HM/HGI (*n* 109)		HM/LGI (*n* 112)	
Fatty acid subclass	Mean	sd	Mean	sd	Mean	sd	Mean	sd
SFA								
Baseline (post-run-in)	15·9	10·4	15·1	9·0	14·4	7·7	15·8	10·0
Post-intervention	15·5	10·3	15·3	9·7	15·4	9·8	15·2	9·6
Change	−0·4	10·7	+0·2	9·26	+1·0	7·8	−0·6	10·5
MUFA								
Baseline (post-run-in)	4·2	2·8	3·9	2·4	3·7	2·0	4·1	2·7
Post-intervention	4·3	3·2	4·3	2·9	4·3	2·9	4·4	2·8
Change	+0·1	3·1	+0·42	2·6	+0·6	2·2	+0·3	3·0
*n*-3 PUFA								
Baseline (post-run-in)	3·1	2·5	2·9	2·0	2·7	1·9	3·1	2·1
Post-intervention	2·9	2·4	2·8	1·9	2·8	2·1	2·8	1·8
Change	−0·2	2·6	−0·1	1·9	0·1	1·7	−0·3	2·0
*n*-6 PUFA								
Baseline (post-run-in)	12·6	8·8	11·8	7·4	11·4	6·4	12·2	8·3
Post-intervention	12·4	9·4	12·2	8·2	12·2	8·3	12·2	8·1
Change	−0·2	9·6	+0·3	7·7	+0·7	6·6	−0·0	8·7
Total PUFA								
Baseline (post-run-in)	15·7	10·9	14·7	9·1	14·1	7·9	15·3	10·1
Post-intervention	15·3	11·7	15·0	9·9	14·9	10·1	15·0	9·7
Change	−0·4	11·9	+0·2	9·4	+0·7	8·0	−0·3	10·5

HM, high MUFA; HGI, high glycaemic index; LGI, LGI, low glycaemic index.

In HM groups, increases in plasma MUFA (%NEFA_total_) were associated with positive changes in Si (*r* 0·218; *P* = 0·001). Bootstrap 95 % CI (based on 1000 samples) were 0·108 and 0·325 for this correlation, which also remained significant when adjusting for change in dietary GI (*r* 0·212; *P* = 0·003) and when a linear regression model adjusting for age, sex, ethnicity and change in body weight was applied (β = 0·233; *P* = 0·001) (Table 3). Additionally, cross-validation was used. The HM dataset was split into 80 %:20 % (training:validation) sets. A stepwise linear regression model which incorporated age, sex, ethnicity and change in body weight was applied (Table 3). Age and change in plasma MUFA (%NEFA_total_) were significant interacting variables. Comparable Pearson's correlation coefficients were attained when assessing predicted *v.* actual change in Si between the two sets (*r* 0·266 and *r* 0·275, respectively), although the validation set did not reach significance (*P* = 0·068), probably owing to small sample size.

**Table 3.** Results of multiple regression analysis assessing the relationship between the change in plasma MUFA concentration (as a percentage of the total plasma phospholipid fatty acid pool) and changes in insulin sensitivity index (Si), according to study group, controlling for age, sex, ethnicity, change in body weight and changes in SFA and PUFA levels (as a percentage of the total plasma phospholipid fatty acid pool)

Group	Dependent variable	Independent variable	Control variables	β Coefficient	se	Standardised β coefficient	*R*^2^	*R*^2^ change	VIF	*P*
HM	Si	Change in MUFA (% of total NEFA)	Age, sex, ethnicity, change in body weight	0·040	0·012	0·233	0·041	0·054	1·1	0·001
HM/LGI	Si	Change in MUFA (% of total NEFA)	Age, sex, ethnicity, change in body weight	0·059	0·017	0·321	0·094	0·099	1·1	0·001
HM	Si	Change in MUFA (% of total NEFA)	Change in SFA (% of total NEFA)	0·085	0·036	0·475	0·034	0·025	9·2	0·020
HM	Si	Change in MUFA (% of total NEFA)	Change in PUFA (% total NEFA)	0·039	0·040	0·224	0·048	0·048	12·1	0·006
HM (80 % training sample)	Si	Change in MUFA (% of total NEFA)	Age, sex, ethnicity, change in body weight	0·037	0·014	0·203	0·061	0·035	1·1	0·007

HM, high MUFA; LGI, low glycaemic index; VIF, variance inflation factor.

Where VIF is provided for multiple control variables it specifies the highest VIF value for the variables analysed within the model.

Binary logistic regression was performed to assess the relationship between increasing proportion of PL MUFA (%NEFA_total_) and the likelihood of an improvement in Si across the study period. Change in Si was categorised as positive and negative. A model including, age, sex, ethnicity and change in body weight, within HM diet groups, was applied (Table 4). The model correctly identified 67 % of cases. Increases in MUFA (%NEFA_total_) were positively associated with probability of increases in Si over the study period (*B* = 0·204; 95% CI 1·077, 1·397; *P* = 0·002).

**Table 4.** Result of binary logistic regression analysis assessing the relationship between change in plasma phospholipid MUFA concentration (as a percentage of the total plasma fatty acid pool) and change in insulin sensitivity index categorised as positive and negative for subjects receiving high-MUFA diets* (Unstandardised coefficients (*B*) with their standard errors; odds ratios and 95% confidence intervals)

Variable	*B*	se	OR	95 % CI	*P*
Age	−0·032	0·015	0·968	0·941, 0·996	0·027
Sex	0·490	0·295	1·632	0·916, 2·909	0·970
Ethnicity (categorised variable)	−0·298	0·208	0·742	0·494, 1·115	0·151
Change in body weight	−0·128	0·069	0·880	0·769, 1·007	0·062
Change in MUFA (% of total NEFA)	0·204	0·066	1·227	1·077, 1·397	0·002

* Covariates included: age, sex, ethnicity (demographic variables), change in body weight.

When HM groups were separated into HM/HGI and HM/LGI groups the association with increasing Si was only significant in the low-GI group (*r* 0·297; *P* = 0·002). After controlling for change in GI, this association remained significant (*r* 0·303; *P* = 0·002). The relationship also remained after adjustment for age, sex, ethnicity and change in body weight (β = 0·321; *P* = 0·001).

In the HM groups, change in oleic acid (18 : 1) was most strongly positively correlated with change in Si (*r* 0·204; *P* = 0·003), as was change in minor plasma MUFA 24 : 1 (*r* 0·180; *P* = 0·008), *trans-*18 : 1 (*r* 0·150; *P* = 0·035) and 17 : 1 (*r* 0·145; *P* = 0·035). Similarly, in the HM/LGI group, change in oleic acid (18 : 1) was most strongly correlated with change in Si (*r* 0·266; *P* = 0·005), as was 24 : 1 (*r* 0·244; *P* = 0·011) and 17 : 1 (*r* 0·196; *P* = 0·042). These associations remained significant after adjustment for change in GI. When incorporating 24 : 1, *trans*-18 : 1 and 17 : 1 into a stepwise regression model there was no significant additive effect on change in SI when compared with oleic acid alone.

No association was observed between change in palmitoleic acid and change in Si when HM groups were considered together or individually. It may also be noted that within HM groups, changes in total SFA and PUFA (%NEFA_total_) were positively associated with change in Si (*r* 0·184, *P* = 0·007; *r* 0·208, *P* = 0·002, respectively). However, the positive relationship between percentage change in MUFA (%NEFA_total_) and change in Si remained when the effects of change in SFA (%NEFA_total_) were controlled for (Table 3). The effect of changes in PUFA concentrations of Si could not be accurately controlled for due to excessive collinearity with MUFA concentration (Table 3).

There was no statistical association between reported change in the average dietary oleic acid concentration calculated from nutritional diaries within the HM group across the study period and change in plasma PL oleic acid concentration (*r* −0·007; *P* = 0·918) (even when dietary oleic intake was expressed per kJ).

## Discussion

Original analysis of data from the RISCK study indicated that the replacement of dietary saturated fat with monounsaturated fat as a result of dietary intervention did not result in a significant improvement in insulin sensitivity^(11)^. However, it has not previously been considered how changes in the proportion of individual FA and FA subclasses may correspond with changes in insulin sensitivity. Our re-assessment of the RISCK dataset suggests that increases in the proportion of the plasma FA pool comprising monounsaturated PL FA (most notably oleic acid) in subjects who consumed a HM dietary intervention were associated with a modest improvement in insulin sensitivity.

There is conflicting evidence from randomised controlled trials on the effect of dietary fat composition on insulin sensitivity, and the potential benefits of HM diets. The KANWU study, a 3-month dietary intervention study similar in design to RISCK, noted improvements in Si following a reduction in the proportion of dietary saturated NEFA and a corresponding increase in MUFA^(5)^. This finding was replicated by a smaller intervention study that compared the effects of a HM diet and high-saturated fat diet on insulin sensitivity^(4)^ but not by the results of RISCK or another large pan-European dietary intervention study, LIPGENE; both failed to demonstrate a significant change on Si following isoenergetic replacement of high-saturated fat diets with HM or low-fat/high-carbohydrate diets^(6)^. This highlights the current uncertainty regarding the optimal dietary strategy for T2DM prevention.

Previous studies have suggested a link between high-saturated fat diets (and serum SFA content) and increased progression to impaired fasting glycaemia and T2DM^(14,15)^. However, there is also evidence that individual FA differentially influence insulin sensitivity^(7)^. A recent large-scale European cohort study focusing of SFA demonstrated that individual FA were associated both positively and negatively with the incidence of T2DM^(16)^. This further suggests it may be an oversimplification to consider an entire FA class as protective or deleterious with respect to T2DM risk and demonstrates the importance of considering the individual effects of NEFA. At baseline there was a weak inverse association between total saturated fat concentration and Si, consistent with the literature. While there was an inverse correlation between baseline total *n*-6 PUFA and Si in the RISCK dataset this finding is less consistent with the body of research that generally indicates that a diet lacking essential *n*-6 PUFA such as linoleic acid may promote insulin resistance^(17)^. Further dietary intervention studies with sustained high PUFA consumption are required to fully establish the metabolic effects of PUFA on Si.

The fact that total, saturated and unsaturated NEFA concentration at baseline were inversely correlated indicates that, regardless of fat type, if total consumption (and therefore plasma fat concentration) increases beyond a point, insulin sensitivity deteriorates, as is the consensus view. Interestingly, the KANWU study only reported an improvement in insulin sensitivity following substitution of SFA for MUFA when fat consumption contributed <37 % of total energy intake^(5)^. This would imply that provided fat consumption is kept within recommended limits, manipulation of dietary fat type to ensure higher proportions of certain fat subgroups, such as MUFA, may benefit insulin sensitivity. Modification of dietary fat quality as opposed to quantity may be a more achievable strategy at a public health level given that current efforts aimed at reducing total fat consumption have been widely unsuccessful.

Although changes in PL MUFA concentrations correlated with improvements in insulin sensitivity in HM groups, and original RISCK analysis demonstrated that dietary intervention achieved significant increases in PL MUFA levels in these groups, our results failed to show a relationship between reported oleic acid intake as a proportion of total energy intake and change in plasma levels. The explanation for this may lie in the relative contribution of dietary FA intake to the various lipid fractions and the representation of a specific FA within different FA moieties. Additionally, certain endogenous FA may be more useful biomarkers of FA intake compared with others. It has been previously shown that the relationship between reported FA intake and changes to cholesteryl ester FA which is demonstrable for major SFA, certain *trans*-FA and linoleic is absent for oleic acid; thus it has been suggested that oleate has limited utility as a marker of quantitative biomarker dietary intake in observational studies^(18)^. Furthermore, a systematic review by Hodson *et al*.^(19)^ also noted that the magnitude of response of the PL FA pool to changes in dietary FA composition is considerably weaker compared with cholesteryl esters and TAG. Thus, measurement of FA concentrations within other constituents of the plasma lipid pool may be more sensitive to smaller changes.

The optimal dietary strategy for the prevention of T2DM with regard to fat consumption remains uncertain. Our data suggest a modest benefit of increasing the proportion of dietary monounsaturated fat (most significantly the major MUFA, oleic acid) on insulin sensitivity. Longer-term interventions would be required to assess the absolute risk reductions for T2DM in populations adhering to this characteristically ‘Mediterranean’ dietary model, replete in MUFA sources such as olive oil. On the basis of current evidence, however, dietary recommendations for T2DM prevention are likely to mirror those for CVD prevention: reducing total saturated fat intake to <10 % and substituting saturated fat sources for unsaturated fats where possible^(20)^.

The strength of these data is that changes in Si have been assessed against changing NEFA levels following a long (6-month) intervention period. However, a limitation of the study is that changes in levels of plasma PL FA were measured, which are more likely to fluctuate in response to short-term changes in dietary intake when compared with erythrocyte membrane FA for example, which would more accurately reflect longer-term changes in dietary fat over months^(21)^. Grouping of NEFA may also overlook the differing effects of individual FA, whose physiological roles will be influenced by their carbon chain lengths. Furthermore, data on physical activity were unavailable; hence the effects of exercise on NEFA levels could not be adjusted for. Since the study was conducted solely in individuals at elevated metabolic risk, generalisability is limited. However, the effects of NEFA and dietary modification in T2DM prevention are most pertinent in this high-risk group.

The limitation of our analysis is that it is a subanalysis of a randomised study where the aim was to compare insulin sensitivity across the low and high MUFA and GI diets. We believe that this analysis does suggest that there is a relationship between plasma MUFA, which may be reflective of individual compliance with the diet or individually different metabolism of MUFA, and insulin sensitivity.

Progression from euglycaemia to T2DM is usually a gradual process over time of β-cell dysfunction and of declining insulin sensitivity. Small dietary changes over many years may confer substantial benefit in slowing this progression. At a population level, substituting ‘high-risk’ sources of dietary fat for those that may have a small protective effect, such as MUFA, may help to combat the rapid rise in T2DM prevalence.

## Supplementary material

For supplementary material accompanying this paper visit https://doi.org/10.1017/jns.2019.29. click here to view supplementary material
