# Supplementary material for: Increasing the proportion of plasma MUFA, as a result of dietary intervention, is associated with a modest improvement in insulin sensitivity
Source: J Nutr Sci. 2019 Nov 29;9:e6. doi: 10.1017/jns.2019.29 (PMC7003243; doi:10.1017/jns.2019.29)
Supplement: Supplementary file 1 [file S2048679019000296sup001.zip › JNS1900029_JOHNS_Supplementary_Table_S3.docx]

**Supplementary Table S3.** Full list of plasma phospholipid fatty acids analysed with fatty acid nomenclature

| Fatty acid | Fatty acid class | *n-x* (or omega-x; location of 1st double bond) and cis/trans isomerism | Lipid number |
| --- | --- | --- | --- |
| Butyric | Saturated |  | 4 : 0 |
| Caproic | Saturated |  | 6 : 0 |
| Caprylic | Saturated |  | 8 : 0 |
| Capric | Saturated |  | 10 : 0 |
| Undecanoic | Saturated |  | 11 : 0 |
| Lauric | Saturated |  | 12 : 0 |
| Tridecanoic | Saturated |  | 13 : 0 |
| Myristic | Saturated |  | 14 : 0 |
| Myristoleic | MUFA | n-5 (cis) | 14 : 1 |
| cis-10-pentadecanoic | MUFA | n-10 (cis) | 15 : 1 |
| Palmitic | Saturated |  | 16 : 0 |
| Palmitoleic | MUFA | n-7 (cis) | 16 : 1 |
| heptadecanoic | Saturated |  | 17 : 0 |
| cis-10heptadecanoic | MUFA | n-7 (cis) | 17 : 1 |
| Stearic | Saturated |  | 18 : 0 |
| Elaidic | MUFA | n-9 (trans) | 18 : 1 |
| Oleic | MUFA | n-9 (cis) | 18 : 1 |
| Linolelaidic | PUFA | n-6 (all-trans) | 18 : 2 |
| Linoleic | PUFA | n-6 (all-cis) | 18 : 2 |
| Arachidic | Saturated |  | 20 : 0 |
| γ-linolenic | PUFA | n-6 (all-cis) | 18 : 3 |
| cis-11-eicosenoic | MUFA | n-9 (cis) | 20 : 1 |
| Linolenic | PUFA | n-3 (all-cis) | 18 : 3 |
| heneicosanoic | Saturated |  | 21 : 0 |
| cis-11,14-eicosadienoic | PUFA | n-6 (all-cis) | 20 : 2 |
| Behenic | Saturated |  | 22 : 0 |
| cis-8,11,14-eicosatrienoic | PUFA | n-6 (all-cis) | 20 : 3 |
| Erucic | MUFA | n-9 (cis) | 22 : 1 |
| cis-11,14,17-eicosatrienoic | PUFA | n-3 (all-cis) | 20 : 4 |
| Arachidonic | PUFA | n-6 (all-cis) | 20 : 4 |
| Tricosanoic | Saturated |  | 23 : 0 |
| docosadienoic | PUFA | n-6 (all-cis) | 22 : 2 |
| EPA | PUFA | n-3 (all-cis) | 20 : 5 |
| Lignoceric | Saturated |  | 24 : 0 |
| Nervonic | MUFA | n-9 (cis) | 24 : 1 |
| DPA | PUFA | n-3 (all-cis) | 22 : 5 |
| DHA | PUFA | n-3 (all-cis) | 22 : 6 |
