# Supplementary material for: Increasing the proportion of plasma MUFA, as a result of dietary intervention, is associated with a modest improvement in insulin sensitivity
Source: J Nutr Sci. 2019 Nov 29;9:e6. doi: 10.1017/jns.2019.29 (PMC7003243; doi:10.1017/jns.2019.29)

**Supplementary Table S2.** Dietary regimens and targets for fat and carbohydrate consumption as a percentage of total energy intake


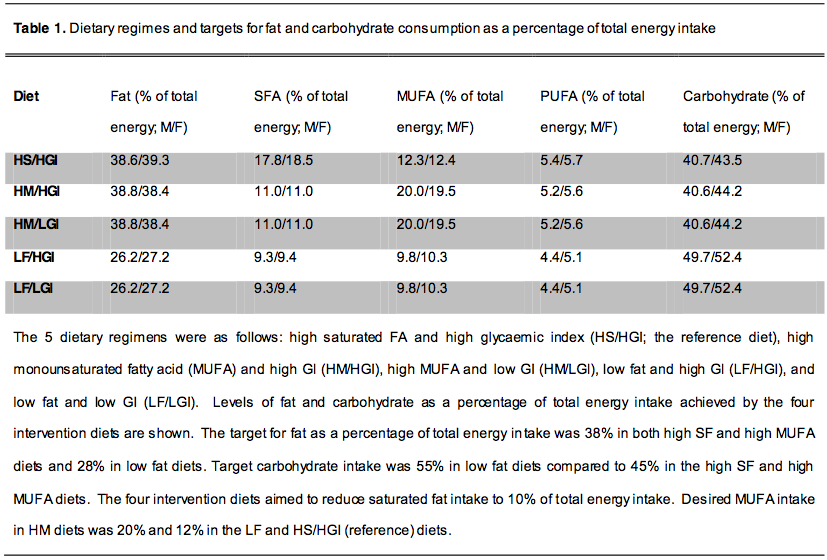

Supplement: Supplementary file 1 [file S2048679019000296sup001.zip › JNS1900029_JOHNS_Supplementary_Table_S2.docx]
