# Supplementary material for: Increasing the proportion of plasma MUFA, as a result of dietary intervention, is associated with a modest improvement in insulin sensitivity
Source: J Nutr Sci. 2019 Nov 29;9:e6. doi: 10.1017/jns.2019.29 (PMC7003243; doi:10.1017/jns.2019.29)
Supplement: Supplementary file 1 [file S2048679019000296sup001.zip › JNS1900029_JOHNS_Supplementary_Table_S1.docx]

**Supplementary Table S1**

*Points system for selection of subjects at elevated risk of metabolic disease:*

Fasting glucose concentration >5.5 mmol/l or insulin concentration >40 pmol/l = 3 points; body mass index (BMI; in kg/m^2^) >30 or waist >102 cm for men and >88 cm for women = 2 points; BMI of 25–30 or waist >94 cm for men and >80 cm (women) = 1 point; treated hypertension = 2 points; systolic BP >140 mm Hg = 1 point; diastolic BP >90 mm Hg = 1 point; HDL-cholesterol concentration <1.0 mmol/l for men and <1.3 mmol/l for women = 2 points; and serum TAG concentration >1.3 mmol/l = 1 point (11). A score of ≥ 4 qualified entry into the study.

*Exclusion criteria*

The exclusion criteria for recruitment of participants to RISCK were as follows: history of ischaemic heart disease; 10-year CVD risk >30%; diabetes mellitus, cancer, pancreatitis, cholestatic liver disease, renal disease; use of lipid lowering medication; systemic corticosteroids, androgens, phenytoin, erythromycin, drugs for regulation of haemostasis (excluding aspirin); exposure to an investigational agent ≤30 days prior to the study; gastrointestinal disorders or use of drugs affecting gastrointestinal motility/absorption; history of alcoholism or substance misuse; current or planned pregnancy or birth in past 12 months; allergy to or intolerance of intervention foods; unwillingness to adhere to study protocol or to provide informed consent; weight change of >3kg in the two months before the study; intake of >1 g eicosapentanoic acid and docosahexaenoic acid/acids; smoking >20 cigarettes per day (11).
